# A Novel Tomato Fusarium Wilt Tolerance Gene

**DOI:** 10.3389/fmicb.2018.01226

**Published:** 2018-06-08

**Authors:** Cahya Prihatna, Martin J. Barbetti, Susan J. Barker

**Affiliations:** ^1^School of Agriculture and Environment, Faculty of Science, The University of Western Australia, Crawley, WA, Australia; ^2^Research and Development for Biotechnology, PT Wilmar Benih Indonesia, Bekasi, Indonesia; ^3^The UWA Institute of Agriculture, Faculty of Science, The University of Western Australia, Crawley, WA, Australia

**Keywords:** Fusarium wilt, tolerance, *Solanum lycopersicum*, *Fusarium oxysporum* f. sp. *lycopersici*, tomato transformation, mycorrhizal symbiosis

## Abstract

The reduced mycorrhizal colonization (*rmc*) tomato mutant is unable to form mycorrhiza and is more susceptible to Fusarium wilt compared with its wild-type isogenic line 76R. The *rmc* mutant has a chromosomal deletion affecting five genes, one of which is similar to *CYCLOPS.* Loss of this gene is responsible for non-mycorrhizality in *rmc* but not enhanced Fusarium wilt susceptibility. Here, we describe assessment of a second gene in the *rmc* deletion, designated *Solyc08g075770* that is expressed in roots. Sequence analyses show that *Solyc08g075770* encodes a small transmembrane protein with putative phosphorylation and glycosylation sites. It is predicted to be localized in the plasma membrane and may function in transmembrane ion transport and/or as a cell surface receptor. Complementation and knock-out strategies were used to test its function. Some putative CRISPR/Cas-9 knock-out transgenic events exhibited Fusarium wilt susceptibility like *rmc* and some putative complementation lines were 76R-like, suggesting that the tomato *Solyc08g075770* functions in Fusarium wilt tolerance. This is the first study to demonstrate that *Solyc08g075770* is the contributor to the *Tfw* locus, conferring tolerance to Fusarium wilt in 76R which was lost in *rmc.*

## Introduction

Fusarium wilt disease in tomato, caused by *Fusarium oxysporum* f. sp. *lycopersici* (Sacc.) W.C. Snyder and H.N. Hans (*Fol*), is one of the most devastating diseases of tomato and has caused major losses in tomato production worldwide. The disease is characterized by wilting and browning of the leaves, yellowing, stunted growth, and eventual death of the plant. Crop yield is negligible in highly infected tomato plantings.

The interaction between *Fol* and tomato is race-cultivar specific. A classical gene-for-gene relationship has been proposed to underly the interaction between *Fol* races and their host cultivars, based on observations of dominant monogenic resistance triggered by challenge with known *Fol* races (Takken and Rep, 2010). Four resistance genes that confer resistance to known *Fol* races have been described in tomato: *I*; *I-2*; *I-3;* and *I-7* (Bohn and Tucker, 1939; Stall and Walter, 1965; Simons et al., 1998; Catanzariti et al., 2015; Gonzalez-Cendales et al., 2016; Catanzariti et al., 2017). Three of these R genes have been incorporated into cultivated tomato from wild tomato species: the *I* and *I-2* genes from *S. pimpinellifolium* that confer resistance to *Fol* races 1 and 2, respectively, and the *I-3* gene from *S. pennellii*, which confers resistance to *Fol* race 3. In addition to the *I-3* gene, resistance to *Fol* race 3 is also conferred by the *I-7* gene (Gonzalez-Cendales et al., 2016). Moreover, *I-7* also confers resistance to *Fol* races 1 and 2. It has been shown that *Avr1* in *Fol* race 1 suppresses the *I-2*- and *I-3*-mediated resistance (Houterman et al., 2008). Resistance to *Fol* race 1 indicates that *I-7-*mediated resistance, unlike *I-2*- or *I-3-*mediated resistance, is not suppressed by *Avr1*, giving further evidence that *Avr1* does not suppress EDS1 (Enhanced Disease Susceptibility 1)-dependent resistance conferred by the *I* and *I-7* genes (Gonzalez-Cendales et al., 2016). *I-3* is located on chromosome 7 and encodes an S-receptor-like kinase, whereas *I-7* is on chromosome 8 and encodes a leucine-rich repeat receptor-like protein (LRR-RLP) (Catanzariti et al., 2015; Gonzalez-Cendales et al., 2016). The *I* gene has recently been identified and like *I-7* it encodes a membrane-anchored LRR-RLP (Catanzariti et al., 2017).

In addition to major resistance conferred by resistance genes, some levels of tolerance or reduced susceptibility in tomato to *Fol* have been reported. Tolerance to *Fol* race 3 has been observed in heterozygous individuals of a *S. lycopersicum* × *S. pennellii* backcrossed population (Bournival et al., 1989). These individuals show moderate resistance to *Fol* race 3 compared to a population carrying the *I-3* locus that is tightly linked to *Got-2* marker on chromosome 7. This moderate disease resistance is linked to a fragment on chromosome 8 marked by *Aps-2* and the locus linked to this marker is designated as *Tfw* (tolerance to Fusarium wilt).

The *rmc* (reduced mycorrhizal colonization) tomato mutant is unable to form mycorrhizal symbiosis with most AM fungal species (Barker et al., 1998; Gao et al., 2001). In addition, *rmc* is also more susceptible to *Fol* race 3 compared with its wild-type isogenic line 76R (Barker et al., 2005). Therefore, the *rmc* mutation causes a contrasting phenotype of root–fungus interaction: colonization by AM fungi is impaired whereas *Fol* race 3 colonization is enhanced. The mutation is a deletion that disrupts five genes on chromosome 8, one of which shares sequence similarity to the *CYCLOPS* gene (Larkan et al., 2007, 2013). A separate study has confirmed that the *CYCLOPS* homolog is the gene that is responsible for the impaired AMF colonization in *rmc*, but does not influence Fusarium wilt tolerance (Prihatna et al., 2018). Thus, the enhanced susceptibility to *Fol* race 3 in *rmc* could be due to disruption of one of the other four genes. Interestingly, *Rmc* is located in the short arm of chromosome 8 that is linked to *Aps-2* where the improved tolerance to Fusarium wilt (*Tfw*) is also located (Larkan et al., 2007). Thus it is likely that *Tfw* is one of the genes in the *Rmc* locus.

Of the other genes that are disrupted in *rmc*, *Solyc08g075770* drew attention, being expressed in roots of 76R but not in *rmc* (Larkan et al., 2013). The predicted transcript sequence encodes a protein of unknown function. It shares sequence similarity with a predicted exopolysaccharide production negative regulator in *Arabidopsis thaliana* (Mayer et al., 1999). To dissect the genetic component leading to enhanced susceptibility to *Fol* race 3 in the *rmc* phenotype, genetic complementation and CRISPR/Cas9-mediated gene knock-out of *Solyc08g075770* were undertaken. Furthermore, a rapid microscopy technique that allows functional analysis of tolerance in transgenic tomato to Fusarium wilt is described.

## Materials and Methods

### Tomato Genotypes

The tomato *rmc* line was generated by single seed descent from a fast neutron mutagenized population that was identified at the M2 generation (Barker et al., 1998). The parental, wild-type line ‘76R’ (cv. Rio Grande) was obtained from Peto Seed Company, Saticoy, CA, United States. The *rmc* tomato has a mutation spanning five genes on the short arm of chromosome 8 flanked by markers CT88 and EST248494 (Larkan et al., 2007, 2013).

### *Fusarium oxysporum* f. sp. *lycopersici* Races

*Fol* races 1, 2, and 3 were used to test susceptibility of tomato 76R, *rmc*, and transformed lines. *Fol* race 1 (accession no. BRIP 17552a), *Fol* race 2 (accession no. BRIP 16848a), and *Fol* race 3 (accession no BRIP 15362a) were obtained from Queensland Plant Pathology Herbarium (Queensland Department of Agriculture, Fisheries, and Forestry). *Fol* BRIP 15362a originated from Dutton Creek, Bowen, QLD, Australia. *Fol* BRIP 16848a originated from Indooroopilly, QLD, Australia. *Fol* BRIP 17552a originated from Severnlea, QLD, Australia.

### Fusarium Wilt Assay

*Fol* isolates were grown on potato dextrose agar for 10 days at room temperature (∼25°C). The surface of the fungal cultures was then flooded with 5 ml of sterile water containing 0.01% Tween 20^TM^ and surface-scraped using a spatula to collect spores. Potato dextrose broth (200 ml) in 500 ml Erlenmeyer flasks was inoculated with 2 ml of the spore suspension and incubated on a rotary shaker (150 rpm) for 5 days at room temperature. After 5 days, spores were collected by filtering the culture through four layers of miracloth. The spore suspension was then centrifuged at 5,000 × *g*, supernatant was decanted and resuspended with sterile water. Spore concentration was counted using a hemocytometer (Improved Neubauer) and concentration adjusted to 1 × 10^7^ spores ml^-1^ (Catanzariti et al., 2015).

Seeds of tomato 76R and *rmc* were sown and germinated in pasteurized potting mix soil. Fourteen-day-old seedlings were uprooted and roots dipped into spore suspension for 3 min, then returned to soil. Plants were watered twice daily and fertilized weekly using Thrive^TM^ liquid complete fertilizer at the recommended rate. There were 10 replicate plants for each treatment, with each plant transplanted into a separate pot. For each tomato genotype, there were four treatments, i.e., *Fol* race 1, *Fol* race 2, *Fol* race 3, and uninoculated control. Plants were grown in a controlled environment room with 16 h photoperiod (25°C light and 22° dark) at 600 Photosynthetic Photon Flux Density (PPFD) and humidity of 70% night (dark), 80% day (light). The experiments were repeated twice.

Disease was evaluated and scored 6 weeks after inoculation. Disease scoring was on a scale of 0–9 based on Fuchs et al. (1997) with modifications, as follows: 0 – healthy plant; 1 – mild chlorosis on lowest two leaves only, mild wilt; 2 – moderate chlorosis on lowest four leaves, mild wilt; 3 – moderate chlorosis on lowest four leaves and mild necrosis on lower two leaves; 4 – moderate chlorosis on lowest four leaves and moderate necrosis on lower two leaves; 5 – moderate chlorosis on lowest six leaves and moderate necrosis on lower four leaves, severe on leaf two; 6 – moderate chlorosis on lowest six leaves and severe necrosis on four leaves, strong wilt; 7 – high chlorosis on lowest six leaves and severe necrosis on four leaves, strong wilt; 8 – plant almost dead, necrosis on all leaves and uppermost crown is chronically wilted; 9 – dead stump. The disease ranking is an ordered categorical (ordinal) variable that has non-Gaussian distribution, thus the statistical model applied was non-parametric. A generalized linear model was used to test the comparison of disease rankings between genotypes and/or *Fol* treatments. Probability was further adjusted using Tukey’s HSD *post hoc* test. Statistical analysis was performed using the R statistics program (R Core Team, 2016). Fresh biomass of plants, crown and leaves were removed from soils and weighed. Statistical analyses were performed using R statistics program and significance of differences in biomass between genotypes and *Fol* races was assessed by two-way ANOVA (*P* < 0.05).

### T-DNA and Vector Constructions

The *Solyc08g075770* protein-coding sequence was cloned into pEarleyGate 100 (Earley et al., 2006) binary vector via Gateway cloning system to generate a binary vector pEarleyGate-Solyc08g075770 (Supplementary Figure S1A). The binary vector clone was kindly donated by Daniel Ruzicka (Donald Danforth Plant Science Center, St. Louis, MO, United States). The *Solyc08g075770* coding sequence is driven by a CaMV35S promoter with duplicated enhancer region (2 × 35S). The vector harbors Basta^®^ resistance gene (*bar*) for transgenic plant selection and kanamycin resistance gene cassette (*nptII* and *KanR*) for bacterial selection.

For genome editing using CRISPR/Cas9, single-guide RNAs (sgRNAs, or protospacers) were designed from genomic sequence of *Solyc08g075770* gene using Benchling, an online molecular biology suite^1^. Construction and cloning of sgRNA into a binary vector was conducted based on methods described by Schiml et al. (2014). The sgRNA was cloned into an entry vector pEn-C1.1 using *Bbs*I restriction site so that it was inserted between U6-26 promoter and sgRNA scaffold. The U6-26 promoter:sgRNA:sgRNA scaffold cassette was then cloned using Gateway Cloning system into a destination vector pDe-CAS9 to generate a binary vector pRMC-CAS9 (Supplementary Figures S1B,C). pEn-C1.1 and pDe-CAS9 were kindly provided by Holger Puchta (Botanical Institute II, Karlsruhe Institute of Technology, Germany) (Schiml et al., 2014).

### Bioinformatics of *Solyc08g075770* Gene

The protein coding sequence (cDNA) of *Solyc08g075770* was translated into protein sequence with the standard genetic code using Translate tool^2^. The resulting protein sequence was used for sequence analyses. The amino acid sequence of Solyc08g075770 was queried in BLAST protein (BLASTP) against non-redundant (nr) protein sequence database containing entries from some members of Solanaceae, Brassicaceae, Fabaceae, Poaceae, Orchidaceae, gymnosperm lycophyte and moss. Protein sequences retrieved from BLASTP hits were downloaded and aligned using Clustal Omega (Sievers et al., 2011) and visualized using JalView (Waterhouse et al., 2009). A phylogenetic tree was built from the aligned sequences and inferred by using Maximum Likelihood method with 500 bootstraps (Jones et al., 1992). These evolutionary analyses were conducted in MEGA7 (Kumar et al., 2016).

Protein sequences from some members of the Solanaceae family were then aligned using Clustal Omega. Structural and functional components were assessed using available bioinformatics tools. Transmembrane helices in Solyc08g075770 were predicted using Hidden Markov Model via TMHMM Server v. 2.0^3^. Homology modeling was attempted using SWISS-MODEL^4^ (Biasini et al., 2014). Template was searched using BLAST against the SWISS-MODEL template library and templates with highest quality selected for model building using ProMod-II (Guex et al., 2009). Overall structure prediction and gene ontology were performed using PSIPRED Protein Sequence Analysis Workbench^5^ and AmiGO 2^6^. Functional motifs and post-translational modifications were predicted using PROSITE^7^.

### Tomato Transformation Procedure

*Agrobacterium tumefaciens* strain AGL1 (Lazo et al., 1991) was used to transform *rmc* tomato for genetic complementation and 76R tomato for CRISPR/Cas9 sequence modification. For transformation, cotyledons of 10-day-old tomato seedlings were cut at the distal and proximal ends and the middle parts were used as transformation explants. The explants were pre-cultured on co-cultivation medium (CCM) for 24 h in the dark at 25°C and then inoculated with AGL1 cell suspension (OD600 = 0.2). The inoculated explants were returned to CCM and incubated for 48 h in the dark at 25°C. After 48 h, explants were washed with timentin solution (300 mg l^-1^) several times to remove bacterial cells and transferred onto pre-selection, shoot induction medium (SIM1) without plant selection and incubated for 7 days at 25°C in white, cool light fluorescence [150 PPFD (μmol m^-2^ s^-1^)]. After a week, explants were transferred to second shoot induction medium (SIM2) which comprised of SIM1 containing 0.5 mg l^-1^ phosphinothricin, to select resistant, transformed cells. Calli and shoots were cultured on this medium for 4 weeks and refreshed onto new medium every week. Healthy calli were isolated from necrotic tissues to prevent cross-poisoning from dying to healthy tissues. At this stage, developed shoots were transferred to shoot elongation medium (SEM) until shoots were about 2.5 cm in length prior to transferring to a rooting medium (RM). Surviving explants and calli were transferred back to SIM1 to recover and regenerate more shoot primordia. Composition of media used for tissue cultures in this study is provided in Supplementary Table S1.

### Evaluation of Putative Transgenic Events

Genomic DNA of regenerated plantlets in tissue culture was analyzed by PCR. Genomic DNA was extracted using a cetyltrimethylammonium bromide (CTAB) method adapted from Murray and Thompson (1980). To detect genetic complementation of *rmc* with *Solyc08g075770*, primers AKCDS4-F and AKCDS4-R were used to amplify 174 bp fragment of *Solyc08g075770* coding sequence in complemented *rmc* and 888 bp fragment of non-mutant (*Rmc*, 76R) genomic DNA. Thus, these primers can differentiate genuine transgenic *rmc* events from 76R contamination. Act2F-Act2R primers were used to amplify the actin gene as a positive PCR control. All PCR was performed using MyTaq^TM^ Red Mix (BIOLINE). PCR conditions were as follows: initial denaturation at 95°C for 3 min followed by 35 cycles of denaturation at 95°C for 30 s, primer annealing at 56°C for 30 s, and polymerisation at 72°C for 50 s. The PCR was finalized by polymerisation at 72°C for 5 min.

To detect CRISPR/Cas9 transgenic 76R events, Cas9 gene was amplified using primers Cas9F1 and Cas9R1. Concomitant with PCR of Cas9, a genomic fragment covering the protospacer region was amplified using primers CRISPR_F1 and CRISPR_R1 and the amplicons were then Sanger-sequenced using the same primers. The *Solyc08g075770* genomic region of 76R was also amplified and sequenced. The sequenced DNA was then analyzed for any mutations.

To ensure there was no genotype contamination, i.e., 76R contamination in the complemented *rmc* and *rmc* in the CRISPR/Cas9 genome editing of 76R, an additional PCR was performed to amplify *CYCLOPS* in all plants used for examination. Hence, *CYCLOPS* is amplified from 76R but not from *rmc*. Primers used to amplify *CYCLOPS* were RmcCDS1S and RmcCDS1A. The sequences of the primers used in this study are provided in Supplementary Table S2.

### Microscopy of Root Colonization

Tissue-culture-grown 76R, *rmc*, and putative transformed plants were uprooted from rooting agar media and the roots washed several times using sterile water to remove agar from the roots. Plants were then put into glass jars containing *Fol* race 3 spore suspension at a concentration of 10^7^ spores ml^-1^ in water. Plants were leaned against the walls of the jars with the roots immersed in spore suspension. One plant from each genotype was put in one jar and there were three replicates for each plant genotype. The experiments were fully repeated twice. The jars were incubated in a growth chamber for 7 days at 25°C in white, cool light fluorescence [150 PPFD (μmol m^-2^ s^-1^)].

Roots were examined for colonization every 12 h using a light compound microscope (Leica DM4000 B LED, Leica Microsystems CMS GmbH). For each observation, at least six roots were cut at approximately 1-cm-length and then stained by ink-vinegar as described in Vierheilig et al. (1998) with some modifications. Root materials were harvested and cleared using 10% (w/v) KOH at 55°C overnight. Cleared roots were rinsed several times with water and then immersed in 0.1 N HCl for approximately 30 s or until the roots appeared slightly lighter in color. Acidified roots were then stained using an ink-vinegar solution [5% (v/v) Parker Quink ink in white vinegar] for 1 h at 55°C followed by 1 h at room temperature. Roots were then destained in a 5% (v/v) vinegar solution for 30 min and washed several times with water and stored in lactoglycerol (lactic acid: glycerol: dH_2_O = 1:1:1, v/v/v). Roots were observed using phase-contrast microscopy with 40×, 100×, 200×, and 400× magnifications. Roots were scanned starting from root tips and then moving away toward distal parts of roots sequentially to span at least 10 overlapping regions of each root.

To quantify the levels of *Fol* colonization on the roots, total area of mycelia was measured and calculated against the total area of each image and expressed as percentage. The analysis was performed using ImageJ (Schindelin et al., 2012). The red–green–blue (true color) channels of images were first split into 8-bit images to obtain grayscale images. To highlight the area of interest, i.e., hyphae/mycelia, the threshold of the images was adjusted and then measurements were performed on the images with parameters delimited by the threshold values.

## Results

### *rmc* Tomato Mutant Is More Susceptible to *Fol* Race 3 Compared to 76R

**FIGURE 1:**
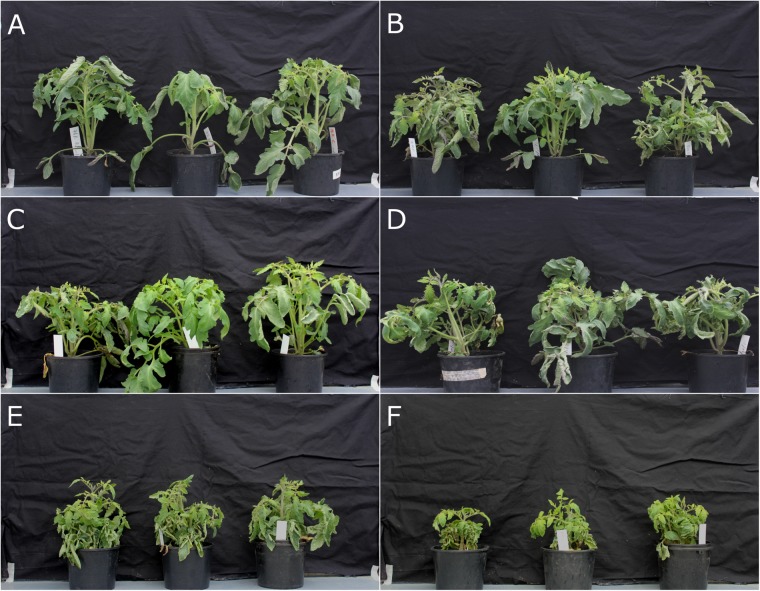
Fusarium wilt disease assay on *rmc* and 76R tomatoes confirming *rmc* as more susceptible to *Fusarium oxysporum* f. sp. *lycopersici* (*Fol*) race 3 than 76R. **(A)** Tomato 76R and **(B)** *rmc* were inoculated with *Fol* race 1; **(C,D)** 76R and *rmc* were inoculated with *Fol* race 2; **(E,F)** 76R and *rmc* were inoculated with *Fol* race 3. Plants were inoculated using a root dip method and photographed 3 weeks after inoculation.

Fusarium wilt bioassay in this study confirmed the results from a previous study that shows *rmc* is more susceptible to *Fol* race 3 compared with 76R but that both tomato genotypes were resistant to *Fol* race 1 (Barker et al., 2005). Early symptoms of wilt appeared at 2 weeks post inoculation (wpi) and were yellowing of older leaves and stunted growth. At three wpi, growth of *rmc* and 76R plants inoculated with *Fol* race 3 was severely stunted compared with non-inoculated controls and plants inoculated with *Fol* race 1 (**Figures 1A–F**). Further, growth of *rmc* inoculated with *Fol* race 3 was also significantly more stunted compared with 76R plants (**Figures 1E,F**).

**FIGURE 2:**
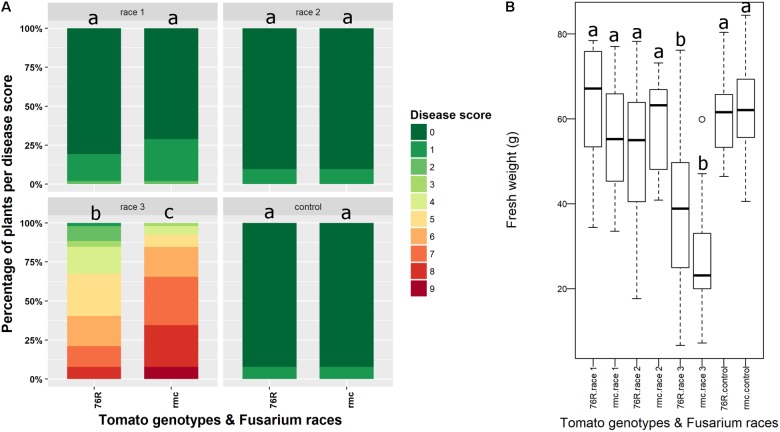
Fusarium wilt disease assessment in *rmc* and 76R 6 weeks after inoculation with *Fusarium oxysporum* f. sp. *lycopersici* (*Fol*) race 1, race 2, and race 3. **(A)** Disease scoring of Fusarium wilt according to Fuchs et al. (1997) at 6 weeks after inoculation. The area for each color code indicates the percentage of plants having the corresponding disease scores. At least 10 plants of either *rmc* or 76R plant type in an individual pot were used in this assay. Control is non-inoculated plants treated only with water. Stacked bars followed by the same letter are not significantly different at the *P* < 0.05 level. **(B)** Box plot analysis showing that fresh weight of *rmc* plants inoculated with *Fol* race 3 is lower than 76R inoculated with *Fol* race 3 although the difference is not significant (*P* > 0.05). Box plots indicated by the same letters are not significantly different at the *P* < 0.05 level.

At six wpi, some *rmc* plants inoculated with *Fol* race 3 had died but no dead 76R plants were observed. Although most of the plants were severely wilted and stunted, *rmc* plants had more severe wilting and necrotic leaves compared with 76R plants (**Figure 2A**). The fresh biomass of *rmc* plants was also lower compared to 76R although the difference was not significant (*P* > 0.05) (**Figure 2B**).

### *Solyc08g075770* Encodes a Small Transmembrane Protein With Predicted Phosphorylation Sites

The predicted protein coding sequence of *Solyc08g075770* gene has 906 base pairs and a search of the tomato genome^8^ identified only one copy of this gene in the tomato genome. It encodes a protein with 301 amino acid sequence with unknown function. The coding sequence was derived from the predicted five exons in the 9.8 kb of gene sequence including the 5′ and 3′ untranslated regions (UTRs) as shown in Supplementary Figure S1C. The gene is located adjacent to a gene that shares sequence similarity to *CYCLOPS* and a predicted ubiquitin-fold modifier 1 gene.

**FIGURE 3:**
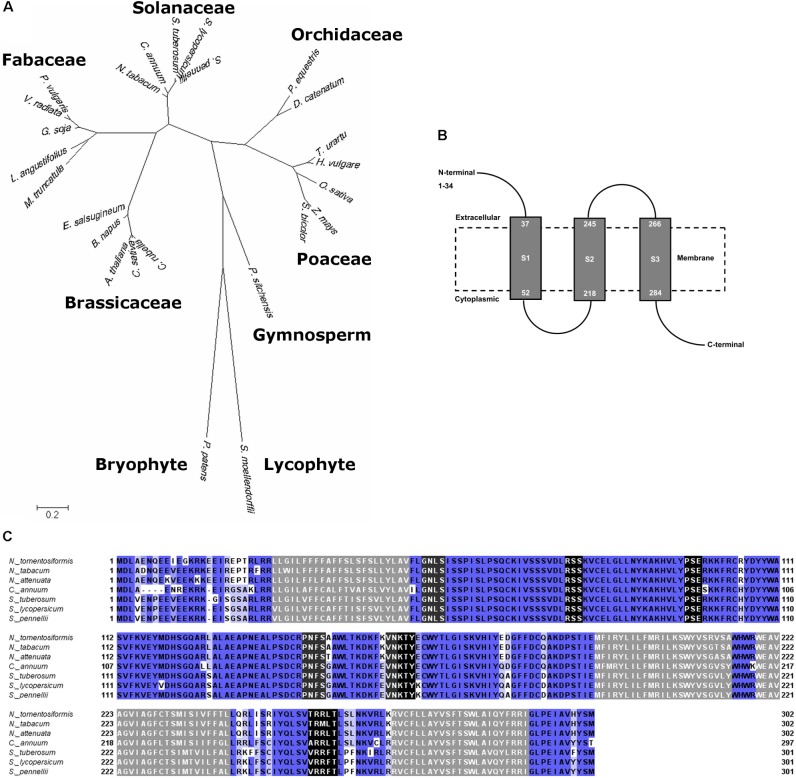
Amino acid sequence analyses of Solyc08g075770. **(A)** Molecular phylogenetic analysis by Maximum Likelihood method of Solyc08g075770 and similar sequences in different plant species with 500 bootstrap replications. The tree is drawn to scale, with branch lengths measured in the number of substitutions per site. **(B)** Solyc08g075770 is predicted to be a protein having three transmembrane domains. Gray boxes are the transmembrane domains, and dashed box is an illustrated cell membrane. **(C)** Multiple alignment of amino acid sequences of Solyc08g075770 and similar sequences in several plants of the Solanaceae family. Sequences that are shaded gray are the predicted transmembrane domains; sequences that are shaded black are phosphorylation sites whereas sequences with dark gray shading are glycosylation sites (*Nicotiana tomentosiformis* XM_009622423, *N. tabacum* XM_016650100, *N. attenuata* XM_019371074, *Capsicum annuum* XM_016717203, *Solanum tuberosum* XM_006364398, *S. pennellii* XM_015227859). Sequences that are shaded blue are sequences that have significant similarity. The intensity of blue color indicates the degree of conservation or sequence identity: the darker the blue the more conserved the sequences.

BLAST search on amino acid sequence of Solyc08g075770 revealed broad sequence similarity across a wide range of plant species including plants of primitive lineages, i.e., the moss *Physcomitrella patens* and the early diverging vascular plant *Selaginella moellendorffii*. The evolutionary tree of Solyc08g075770 in the extant modern plants showed that it has evolved in a distinct pattern from the sequence in extant early land plants, then it further diverged with clear distinction between monocots and dicots (**Figure 3A**).

Solyc08g075770 is predicted to be a membrane-bound protein that contains three transmembrane domains with cytoplasmic (intracellular) glycosylation and kinase phosphorylation sites (**Figure 3B**). There was no nuclear localization signal predicted from the protein sequence. The amino acid sequences are highly conserved amongst Solanaceae plants (**Figure 3C**). Alignment of amino acid sequences in plants that represented dicots (Solanaceae, Brassicaceae, and Fabaceae), monocots (Poaceae and Orchidaceae), gymnosperm, moss, and lycophyte showed less sequence conservation (Supplementary Figure S2). However, some predicted phosphorylation and glycosylation sites were present in all these sequences and this cytoplasmic region is relatively more conserved across the plant species that were assessed. These include phosphorylation motifs V-D-[LIV]-R[SV]-[SA]-K-[VTI]-C and [ERGK]-[TSV]-Y and glycosylation site C-R-P-[NDSVE]-F-[GSAD]. On the other hand, the leading amino acid sequence (N-termini) in the predicted extracellular region was the least conserved, even among the Solanaceae (**Figure 3C** and Supplementary Figure S2). The tomato Solyc08g075770 shared moderate sequence similarity with an exopolysaccharide production negative regulator AT4G19140 in *A. thaliana* chromosome 4 (data not shown) (Mayer et al., 1999). Based on homology modeling, Solyc08g075770 is predicted to have a role in ion transmembrane channel/transport or in a cell surface receptor signaling pathway. Specifically, gene ontology revealed that Solyc08g075770 shared similarity with voltage-gated ion channel and adenylate cyclase-inhibiting G-protein coupled glutamate receptor sequences (Supplementary Table S3).

Search on gene transcripts was conducted to retrieve sequences similar to *Solyc08g075770* that are expressed in roots of tomato induced by *Fol* infection. BLAST search on expressed sequence tag (EST) database in GenBank retrieved four ESTs that were related to *Solyc08g075770* mRNA. These ESTs were FS202019.1, FS189001.1, BI421171.1, and AW441556.1. Of particular interest were ESTs FS202019 and FS189001 that were expressed in roots of tomato Micro-Tom treated with *Fol* race 2 (Aoki et al., 2010). These ESTs (FS202019, cDNA library clone LEFL3149B10; FS189001, cDNA library clone LEFL3062C18) sequences matched the *Solyc08g075770* gene. The EST FS202019 contains the full-length sequence of *Solyc08g075770* mRNA (cDNA library clone LEFL3149B10). The other ESTs, BI421171 and AW441556, were retrieved from EST database of GenBank and these sequences were found in callus and ripe fruit of tomato, respectively (Alcala et al., unpublished).

### Assessment of the Role of *Solyc08g075770* in Tolerance of Tomato to Fusarium Wilt by a Rapid Microscopy Assay

**FIGURE 4:**
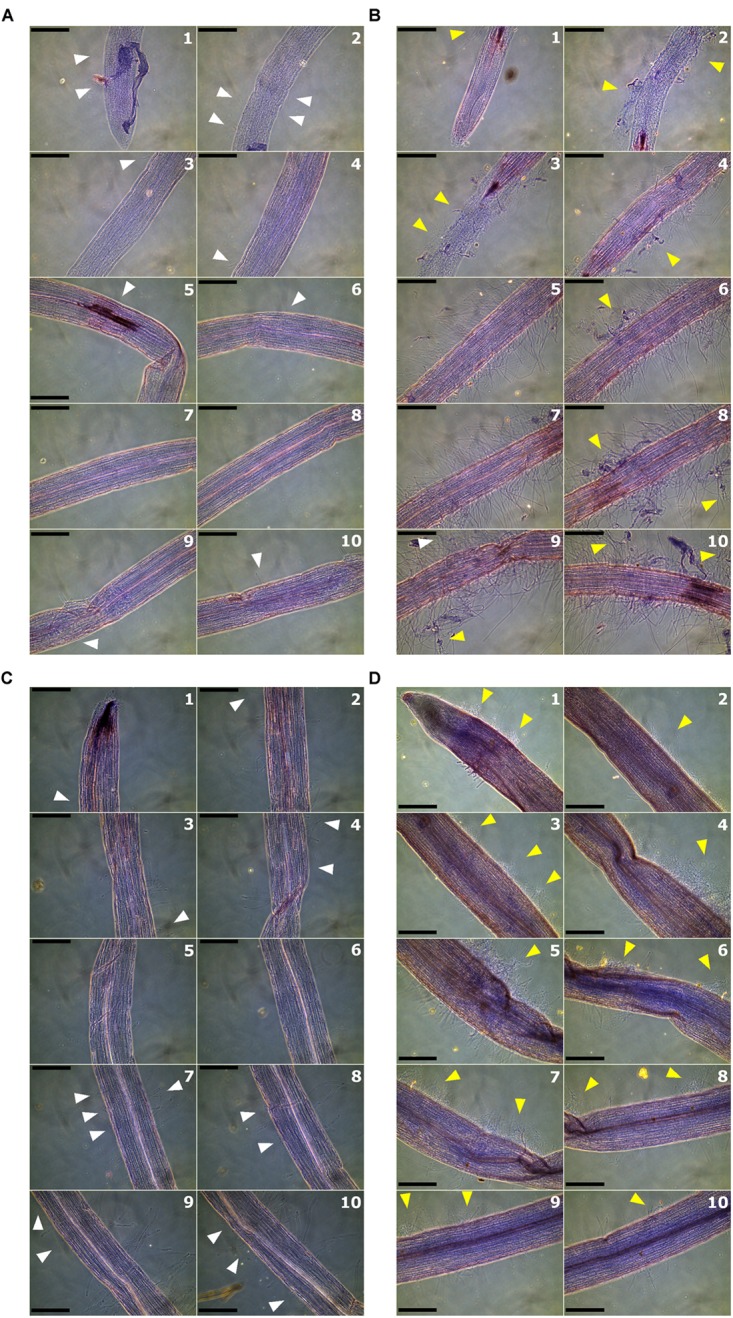
Root colonization by *Fusarium oxysporum* f. sp. *lycopersici* (*Fol*) race 3 observed by phase-contrast microscopy at 24 h after inoculation. Roots were scanned at 10 overlapping zones starting from root tips and moving toward the crown. Numbers in each panel denote the sequential root zones. Light or mild colonization is shown by white arrows whereas heavy colonization is shown by yellow arrows. **(A)** Colonization of roots of 76R; **(B)** root colonization in *rmc*; **(C)** root colonization in 26AK02, a putative complemented *rmc*; and **(D)** root colonization in CRISPR01, a putative knock-out 76R. Scale bars = 200 μm.

**FIGURE 5:**
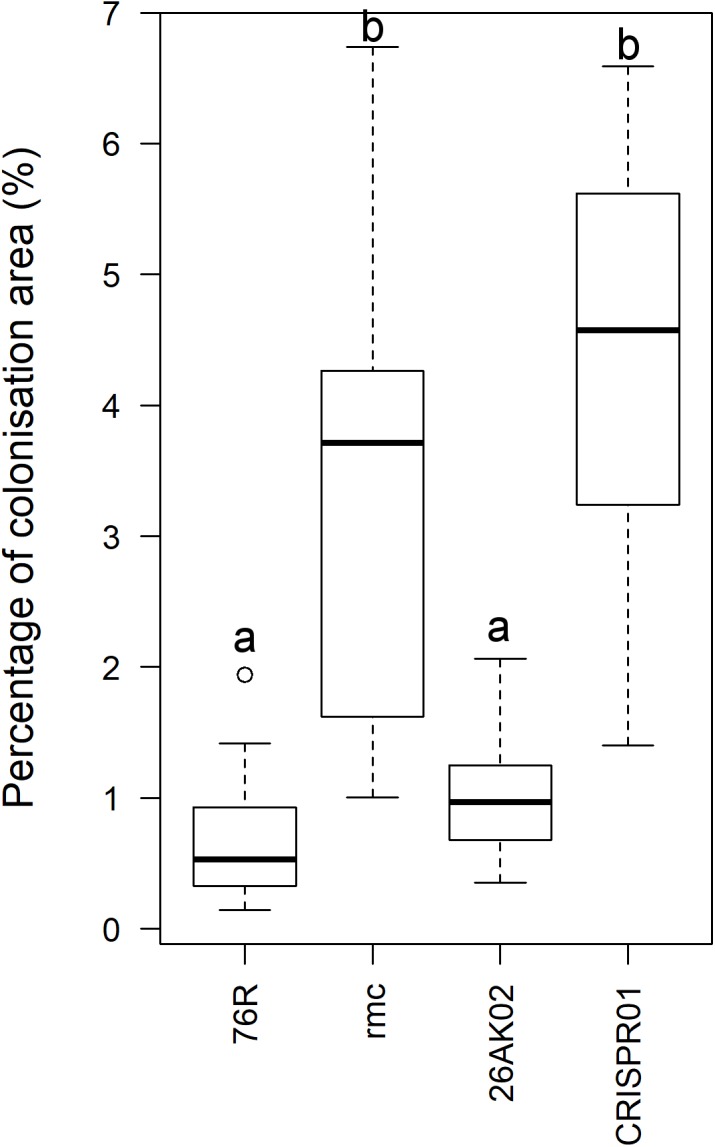
Quantitative analysis of intensity of root colonization by *Fusarium oxysporum* f. sp. *lycopersici* (*Fol*) race 3 observed by phase-contrast microscopy at 24 h after inoculation. Quantitative analysis was performed using ImageJ (Schindelin et al., 2012). Intensity of colonization was measured as area of mycelia against the total area of image. Image quantification was based on gray levels of 8-bit images. Box plot indicated by the same letters are not significantly different (*P* < 0.001).

To determine the role of *Solyc08g075770* gene in tolerance to Fusarium wilt, genetic complementation in *rmc* and gene knock-out in 76R through CRISPR/Cas9 were attempted. There were seven putative complemented *rmc* and 12 putative CRISPR/Cas9 knocked-out mutant events obtained from *Agrobacterium-*mediated transformation. Of these 12 putative CRISPR/Cas9 knocked-out mutants, four did not produce normal shoots and failed to produce enough root materials for testing. Thus, they were discarded, and the remaining eight events were used in this experiment. To facilitate rapid screening of transgenic materials, a microscopy assay was developed that examined the early stages of susceptible Fusarium wilt colonization (*rmc*) compared to Fusarium wilt tolerance (76R). Microscopy scanning on at least 20 roots for each *rmc* and 76R lines revealed that roots of *rmc* were consistently colonized more heavily compared with 76R (**Figures 4A,B**, **5** and Supplementary Table S4). In both 76R and *rmc*, colonization on roots was observed as soon as 12 h after inoculation. At this stage, hyphae were frequently found in the root hairs and less frequently on the root surface. After 24 h, hyphae had colonized root surfaces more intensely and root apex colonization had also commenced. *Fol* race 3 preferentially colonized the elongation and maturation zones of the roots with occasional colonization at the apical meristem. The intensity of colonization gradually decreased toward distal (older) parts of the roots. The difference in the root colonization between 76R and *rmc* was not so much in the frequency of the infection sites, but instead there was a marked difference in the abundance of hyphal growth, with more mass of *Fol* race 3 mycelia on *rmc* roots compared with 76R (**Figures 4**, **5** and Supplementary Table S4).

The extent to which putative complemented *rmc* transgenic events were colonized by *Fol* race 3 was comparable, but not identical, to 76R. Two out of seven putative complemented *rmc* showed the expected phenotype of restored levels of tolerance as in 76R if complementation occurred (events 26AK02 and 26AK11). The other five putative events resembled *rmc*, which is indicative of non-complemented *rmc*. The 26AK11 event resembled lightly colonized *rmc* whereas the 26AK02 resembled slightly heavier colonized 76R (**Figures 4C**, **5** and Supplementary Figure S3). Two out of eight of the putative knock-out mutants (events CRISPR01 and CRISPR02) exhibited *rmc*-like phenotype with heavy *Fol* colonization (**Figures 4D**, **5** and Supplementary Figure S3) indicative of genuine knock-out mutants, whilst other putative knock-out mutant events resembled 76R.

### Molecular Detection of Transformed Tomato Plants

In addition to the rapid microscopy assay, screening of putative transformed plants was conducted by detecting transgene using PCR. PCR amplification of *Solyc08g075770* from several putative complemented *rmc* plants showed either specific but faint signal or non-specific but strong bands of amplicon DNA (Supplementary Figure S4). For the putative complemented *rmc* that showed 76R-like root colonization phenotype (26AK02 and 26AK11), these events also showed relatively stronger bands of amplicon DNA (Supplementary Figure S4A). For CRISPR/Cas9, event analysis Cas9 fragment was amplified from several plants (Supplementary Figure S4). Similarly, the putative knock-out mutants CRISPR01 and CRISPR02, which exhibited *rmc*-like phenotype, showed relatively stronger bands of Cas9 amplicon compared with the other putative mutants (Supplementary Figure S4B). However, the sequence of some putative CRISPR/Cas9 knock-out mutants showed there was no mutation in and around the sgRNA region, the same as in the wild-type 76R (Supplementary Figure S5). These potentially transgenic plants, that had the hall mark control PCR band amplification of their intended parent, were examined for root colonization by *Fol* race 3.

## Discussion

This is the first study to assess the role of the *Solyc08g075770* gene in Fusarium wilt tolerance of tomato. We had hypothesized that the gene is likely involved in root interactions with microbes because of its localized expression in roots and proximal location to a gene that shares similarity to *CYCLOPS* which in other plant species is involved in mycorrhizal symbiosis. We predicted that the *Solyc08g075770* gene encodes a small transmembrane protein containing glycosylation and phosphorylation motifs. Functional analyses through genetic complementation and CRISPR/Cas9 provided supportive evidence of the role of *Solyc08g075770* in Fusarium wilt tolerance. Observation on root colonization by *Fol* race 3 using a rapid microscopy assay highlighted presence of expected phenotypes in complemented *rmc* and knocked-out 76R, further indicating that *Solyc08g075770* functions in tolerance to Fusarium wilt. Together, the results presented in the current study demonstrate that *Solyc08g075770* is the contributor to the *Tfw* locus identified by Bournival et al. (1989), conferring tolerance to *Fol* race 3 in 76R which was lost in *rmc.*

The presence of amino acid coding sequences similar to *Solyc08g075770* in moss and lycophyte genomes suggested that the *Solyc08g075770* has been conserved in evolution since early colonization of land with plants. Unlike the tomato *CYCLOPS*, the *Solyc08g075770* gene is also present in the non-mycorrhizal plant *A. thaliana*. In *A. thaliana*, the gene is predicted to encode an exopolysaccharide production negative regulator (Mayer et al., 1999). Alignment of Solyc08g075770 with several similar sequences in Solanaceae plant genomes allowed the identification of putative transmembrane domains in the Solyc08g075770. Since there is no nuclear localization signal detected, Solyc08g075770 could be an integral component of plasma and/or organelle membranes.

The predicted phosphorylation sites in the cytoplasmic region of Solyc08g075770 suggest that it is a substrate of kinase involved in the signaling pathway. Interestingly, the polymorphic region is located at the leading sequence (N-terminal) which is predicted to be located extracellularly. This suggests that the polymorphic extracellular region could be involved in recognition of biotic or abiotic cues or patterns. However, the lack of putative conserved domains in Solyc08g075770 has made it difficult to predict its function or role in plant–microbe interactions. In particular, Solyc08g075770 lacks the leucine-rich repeat (LRR) and kinase domains (KD) in the LRR-receptor-like kinase (RLK) which are the most widespread protein domains that function in microbial perception and signal transduction (Wei et al., 2015; Liu et al., 2017). Nevertheless, structural modeling predicted that Solyc08g075770 could be an ion channel protein. The role of ion channels in plant immunity has been reported in cyclic nucleotide-gated ion channels (CNGCs) (Moeder et al., 2011). These channel proteins are involved in pathogen-inducible Ca^2+^ influx, which in turn induces reactive oxygen species and mitogen-activated protein kinase (MAPK) cascades in plant defense responses. In higher plants, there are no canonical Ca^2+^ voltage-gated ion channels, but glutamate receptors, together with CNGCs, have been reported (Dietrich et al., 2010). Solyc08g075770 is also predicted to be a G-protein coupled glutamate receptor, suggesting a potential involvement of Solyc08g075770 in Ca^2+^ influx.

Functional analysis of *Solyc08g075770* was determined by microscopic observation on root colonization by *Fol* race 3. The consistent difference in root colonization between *rmc* and 76R indicates that this method provides a rapid and reliable assessment to differentiate the phenotypes between *rmc* and 76R in relation to *Fol* race 3 tolerance or susceptibility. In the traditional disease assay, the more heavily colonized *rmc* roots observed by microscopy correlated with enhanced susceptibility in *rmc* compared with 76R. Results from microscopic observation showed that some putative transformed plants (26AK02, 26AK11, CRISPR01, and CRISPR02) exhibited the expected phenotypes if the plant transformation was successful and thus evidence that *Solyc08g075770* has a role in Fusarium wilt tolerance.

Two putative complemented *rmc* plants (26AK02 and 26AK11) showed light root colonization similar to 76R and two putative knock-out 76R plants (CRISPR01 and CRISPR02) showed heavy colonization similar to *rmc*. However, scanning of roots across at least 10 overlapping regions, starting from root tip then moving away farther to the distal parts of the roots, minimized any chance of random colonization patterns. It was shown that colonization occurred mainly at the elongation and maturation regions of the roots, whereas the root apex, although it was often colonized, was less heavily colonized. Overall, this pattern of colonization is consistent with the findings of Olivain and Alabouvette (1999) who showed that at 24 h after inoculation roots were already colonized mainly at the elongation and mature zone of roots and that the intensity of colonization decreased progressively toward root tip and distal zone. Although internal root colonization was not observed in the current study, the correlation between heavier colonization and increased susceptibility in *rmc* compared with 76R strongly suggests that the extent of root colonization by *Fol* race 3 can predict the levels of tolerance to this pathogen.

The finding of two ESTs (FS202019 and FS189001), representing *Solyc08g075770* in roots of tomato Micro-Tom that were induced by infection of *Fol* race 2, signifies likelihood that this gene is involved in tomato-*Fol* interaction. Furthermore, *rmc* is located on a small region in the short arm of chromosome 8 that is linked to *Aps-2* (Larkan et al., 2007), as is a locus that confers improved tolerance to Fusarium wilt (*Tfw*) (Bournival et al., 1989). The possibility that *CYCLOPS* functions in tolerance to *Fol* has been ruled out in a separate study (Prihatna et al., 2018). Together, results presented in the current study demonstrate that *Solyc08g075770* is the contributor to the *Tfw* locus identified by Bournival et al. (1989), conferring tolerance to *Fol* race 3 in 76R which was lost in *rmc*.

## Author Contributions

CP designed and performed the research, the collection of data, analyzed and interpreted the data, and drafted the manuscript. SB and MB supervised and offered guidance on all aspects of the work and contributed to the writing of the manuscript.

## Conflict of Interest Statement

The authors declare that the research was conducted in the absence of any commercial or financial relationships that could be construed as a potential conflict of interest.

## Footnotes

1 https://benchling.com/

2 http://web.expasy.org/translate/

3 http://www.cbs.dtu.dk/services/TMHMM/

4 http://swissmodel.expasy.org/

5 http://bioinf.cs.ucl.ac.uk/psipred/

6 http://amigo.geneontology.org/amigo

7 http://prosite.expasy.org/

8 https://solgenomics.net/
