# Supplementary material for: A Novel Tomato Fusarium Wilt Tolerance Gene
Source: Front Microbiol. 2018 Jun 8;9:1226. doi: 10.3389/fmicb.2018.01226 (PMC6003170; doi:10.3389/fmicb.2018.01226)
Supplement: Supplementary file 5 [file Image_1.PDF]

Supplementary Material

A Novel Tomato Fusarium Wilt Tolerance Gene

Cahya Prihatna*, Martin John Barbetti, Susan Jane Barker

*** Correspondence:** Cahya Prihatna
cahya.prihatna@research.uwa.edu.au

Supplementary Figures

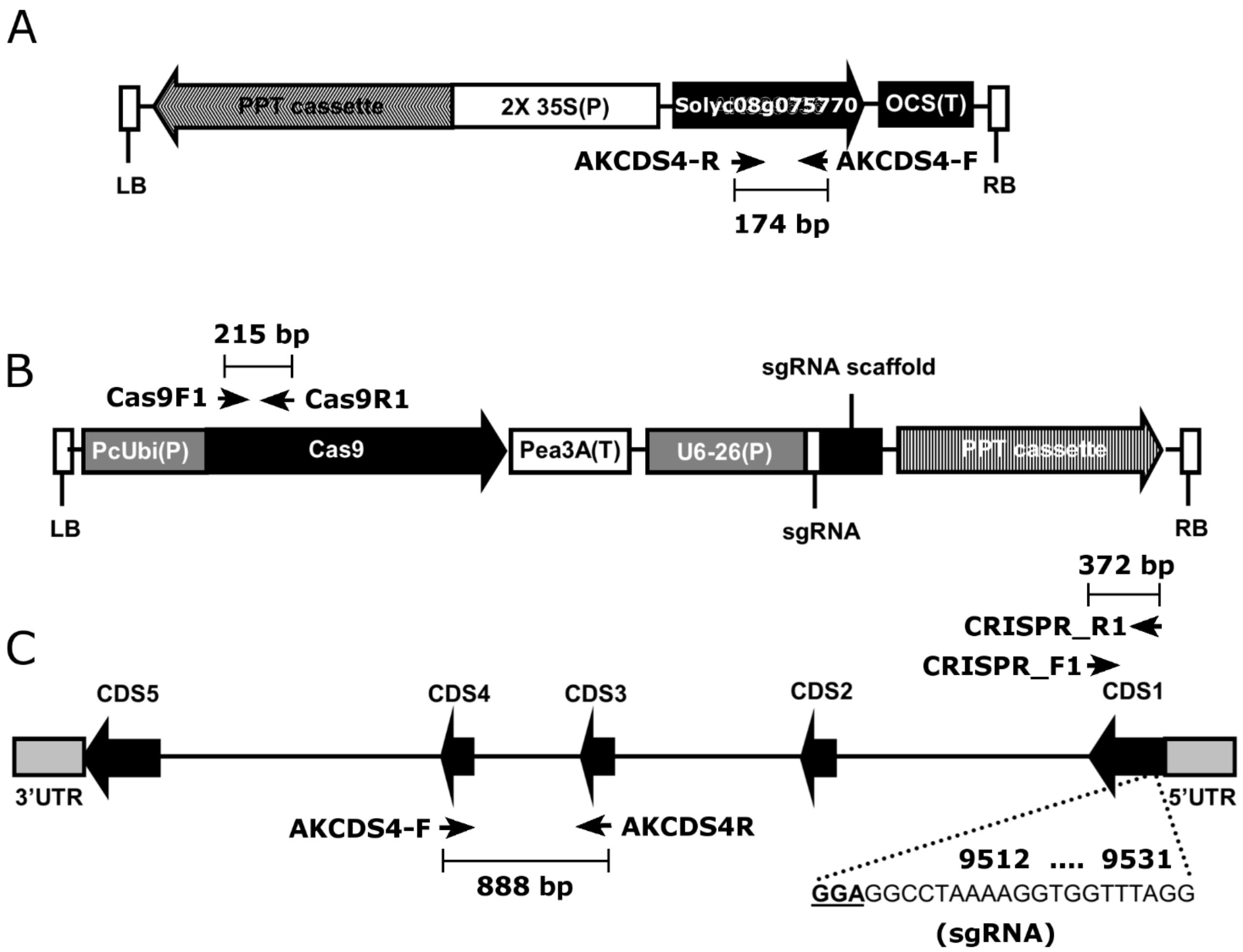

Supplementary Figure 1. T-DNA constructs used in this study and position of guideRNA (protospacer) on Solyc08g075770 gene. A, The construct of Solyc08g075770 coding sequence driven by a CaMV35S promoter with duplicated enhancer region (2×35S) in a binary vector pEarleyGate-Solyc08g075770. B, CRISPR/Cas9 construct in pRMC-CAS9 binary vector. The single-guide RNA (sgRNA) or protospacer is driven by an Arabidopsis U6-26(P) promoter, whereas Cas9 gene is driven by the constitutive ubiquitin 4-2 promoter from Petroselinum crispum (PcUbi4-2). These vectors both carry a selectable marker bar gene that confers resistance to herbicide phosphinotricin. C, The sequence and position of sgRNA on the Solyc08g075770 gene. The sgRNA designed in this study is located on the first exon (CDS1) of Solyc08g075770 at position 9512 to 9531 relative to 3' untranslated transcribed region (3'UTR). Black arrows indicate position of oligonucleotide primers used to amplify fragments of DNA for screening of transgenic plants with the expected size of amplicons is marked by delimiting bars. The names of the primers are shown next to the corresponding arrows.

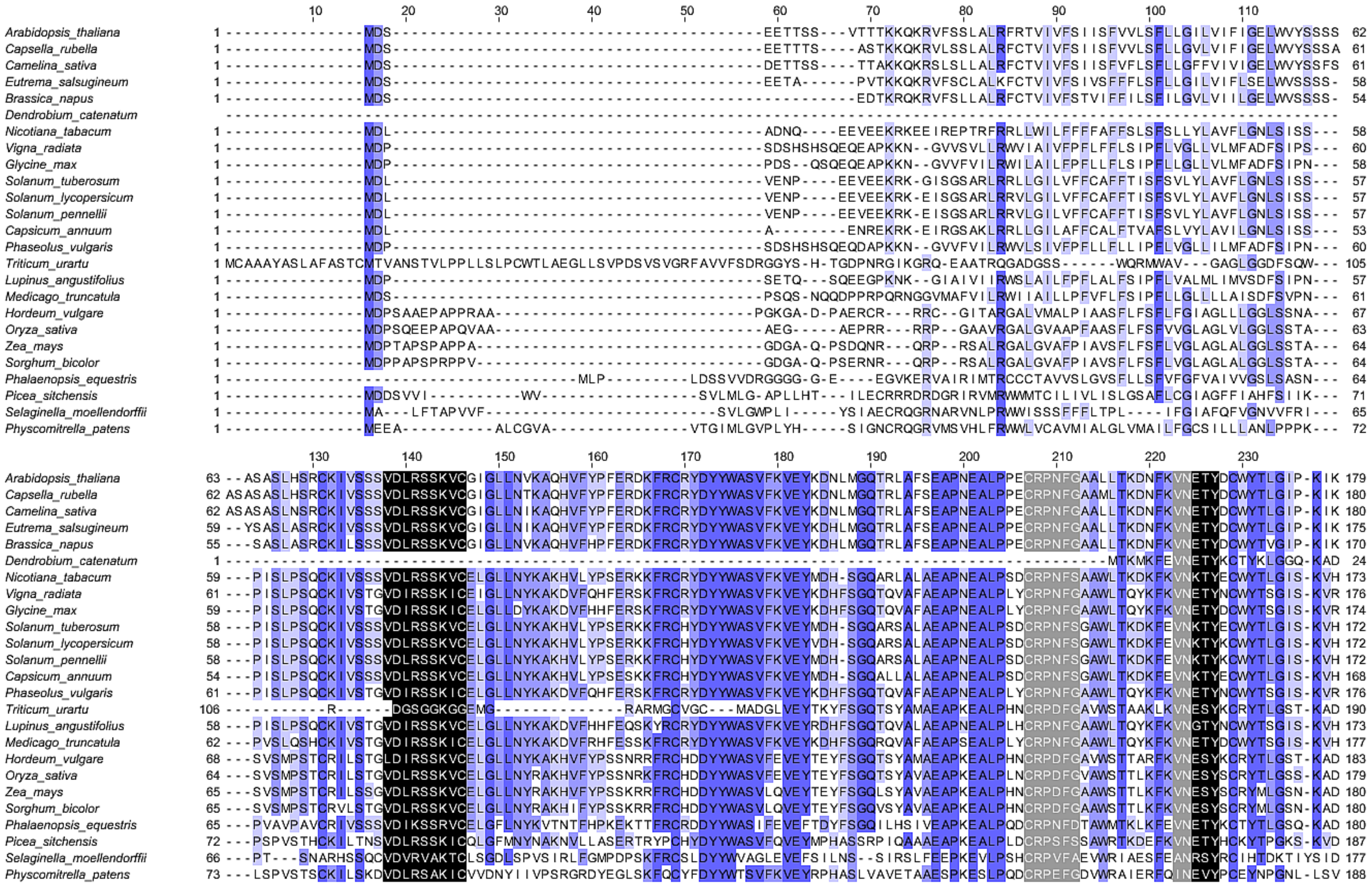
10 20 30 40 50 60 70 80 90 100 110
Arabidopsis_thaliana
Capsella_rubella
Camelina_sativa
Eutrema_salsugineum
Brassica_napus
Dendrobium_catenatum
Nicotiana_tabacum
Vigna_radiata
Glycine_max
Solanum_tuberosum
Solanum_lycopersicum
Solanum_pennellii
Capsicum_annuum
Phaseolus_vulgaris
Triticum_urartu
Lupinus_angustifolius
Medicago_truncatula
Hordeum_vulgare
Oryza_sativa
Zea_mays
Sorghum_bicolor
Phalaenopsis_equestris
Picea_sitchensis
Selaginella_moellendorffii
Physcomitrella_patens
130 140 150 160 170 180 190 200 210 220 230

```
                                      250       260       270       280       290       300       310       320       330       340       350
Arabidopsis_thaliana         180 LYRDSFF-GCQADDLSFTDI-AKQYSIVISKLLQSWLNGEST-PKYWKYDVIAGIVSGFATSIITVFIIRILRHAKSWFPQACCSVKRQFSKVNILVQVKRACLLLVYFSVLGWMATQYL 296
Capsella_rubella             181 LYRDSFF-GCQANDLSFTDI-VKNYSIVISKLVQSWLNGEAT-PKYWKFDVIAGIVSGFATSIITVFIVRILRHAKSWFPQACCSVKNQFSNVNLLIQVKRACLVLVYFSVLGWMAQQYL 297
Camelina_sativa              181 LYRDSFF-GCPANDLSFTDI-VKQYSIVISKLLQSWLNGEAT-PKYWKYDVIAGIVSGFATSMITVFIVRILRHAKSWFPQACCSVKNQFSNVNLLVQVKRACLVLVYFSVLGWMARQYL 297
Eutrema_salsugineum          176 LYQDGFF-GCQANDRSFTDI-FKQYAVLFSRLLQSWFNGKGR-PKYWRYDVIAGIVSGFSTSIITVFVMRILRHAKSWVPRAFCSVKSQYSKVNLVVQMKRACLVLVYFSALGWMATQYL 292
Brassica_napus               171 LYQDGFF-GCQANDLSFTDI-FKQYAVLLSKLLQSWFNGKGR-PKYWRYDVIAGIVSGFSTSIITVFVVRILSNAKSWFPRACCSVKSKLSKVNLVVQVKRACLVIAYFYILGWMATQYL 287
Dendrobium_catenatum          25 IYPDSLF-NCQAKDPSTAEM-VRRFFVLFTRSL-SEKNTAGR----RMGYTVTGIVLGMLTSMFCIILIKILHAAVPSLARQWN--SKKYLI----QSFRRACLLVAYFSAVGLLLLQYK 131
Nicotiana_tabacum            174 IYEDGFF-DCQAKDPSTIEM-FIRYLILFMRILKSWYVSGT-SAWHWRWEAVAGVIAGFCTSMISIVFFALLQRLISRIYQLSV--TRMLTLSLNKVRLKRVCFLLAYVSFTSWLAIQYF 288
Vigna_radiata                177 LPQDNLF-GCHAHQQSTLEK-SREYSTMAMEMAISWFSSRGR-AKHWRWETLIGVVSGFLTSLISITFIRFLHILLSSIYQSFT--TWIFPWRVNTVLVRRACFLIAYLSFVAWLAIEYG 291
Glycine_max                  175 LHQDSLF-GCDAHEQSTLEK-SRQYSTMAMEMVISWFSSRGR-TKHWRWETLAGVVTGFLTSLISITFIRFLQLLLPSLYQSFT--TWIFSWRVNAVLIRRACFLLAYLSFVAWLAIEYG 289
Solanum_tuberosum            173 IYQAGFF-DCDAKDPSTIEM-FIRYLILFMRILKSWYVSGV-LYWHWRWEAVAGVIAGFCTSIMTVILFALLRKFFSCIYQLSV--VRRFTLPFNKIRLRRVCFLLAYVSFTSWLAVQYL 287
Solanum_lycopersicum         173 IYQAGFF-DCDAKDPSTIEM-FIRYLILFMRILKSWYVSGV-LYWHWRWEAVAGVIAGFCTSIMTVILFALLRKLFSCIYQLSV--VRRFTLPFNKVRLRRVCFLLAYVSFTSWLAVQYF 287
Solanum_pennellii            173 IYQAGFF-DCDAKDPSTIEM-FIRYLILFMRILKSWYVSGV-LYWHWRWEAVAGVIAGFCTSIMTVILFALLRKLFSCIYQLSV--VRRFTLPFNKVRLRRVCFLLAYVSFTSWLAVQYF 287
Capsicum_annuum              169 IYQDGFF-DCQAKDPSTIEM-FMRYLILFMRILKSWYVSGV-SYWHWKWEAVAGVIAGFLTSMISIIFFGLLRRLFSCIYQLSV--ARRLTLSLNKVCLRRVCFLLAYVSFTSWLAIQYF 283
Phaseolus_vulgaris           177 LRQDNLF-GCHAHQQSTLEK-SRQYSTMAMEMAISWLSGRGR-TKHWRWETLAGVVSGFLTSLISITFIRFAHILLSSIYQSFT--TWIFPWRVNAVFIRRSCFLLAYLSFVAWLAIEYG 291
Triticum_urartu              191 IYSDKLF-NCTAEDPSLIEM-LKRIFVLFSKFCKSKDFSSWW----MLGYAAAGVVAGMLSSILITVAVRILRGVFLAA-ARR----------------------------------AYS 269
Lupinus_angustifolius        174 LYQDNLF-SCRADEQSNLEK-IKQYSNLALEMLHYWFSNKGS-TKYWKWETIVGVVAGFSTSLISITLLRFLQLLLSSLYRLFA--KSVFPLHVNAVLIRRACLLVAYLSFVAWLAIEYG 288
Medicago_truncatula          178 LYQDNLF-GCRADEQSIIQK-IIQYSTQAMETINYWFSDKGRRANFWRWEIILGVISGFSTSLITITFIMFLKQFLSSLHRPFA--AWILFWRVNAILMKRTCFLIAYSSFVAWLVIVYG 293
Hordeum_vulgare              184 IYSDKLF-NCTAEEPSIIEM-LKRIFVLFSKFCKSKDFSSGR----MLGYAATGVVAGVLSSILITIAVRILRGAFLAA-ARWA--VSNHSMRVFAYRFKRACLLVAYVSFVGWITLQYS 294
Oryza_sativa                 180 IYSDRLF-NCTSEEPSTIEM-LKRIFVLLSKSYMLEDFNSIG----MLGYVMAGVVAGILSALLITILLRSLRGLVLAV-VGSS--VSKHSIRVFACRLKRACLLVAYVSVVGWITLQYS 290
Zea_mays                     181 IHSNKLF-NCTAEEPSTGEL-FKRILILFSEMYVSEDFSSGR----MLVYYVAAGVVLGMLSSMFITALFRGMYGLLLAA-ARWA--VRKHCIRVFASRLKRACLLVAYVSAVGWITLQYS 291
Sorghum_bicolor              181 IHSDKLF-NCTAEEPSTTEL-LKRILILFSEMYVSEDFSSER----MLGYVAAGVVLGMLSSMFITVLFRGFYGLLLAA-ARWA--VRKHSIRAFASRLKRACLLVAYVSAVGWITLQYS 291
Phalaenopsis_equestris       181 IYPDSFF-NCKARDPSTEEM-IRRFFVLFTRSL-SEKSTAGP----QLGFTVAGIVLGMLASLCCIILIKILQAALSSLANQWN--SEKHLI----QILRRACLLVTYLCAFAWLILQWQ 287
Picea_sitchensis             188 IVGDNFF-NCFAKEPSIFEL-IRRVTLLFWHWDQFSFSKKGS-AAHLLWKLVASIFVGMSLSILVACFAKTIRGS--RFIQLGGPDYRKADAASSESRVQFFCICIASLFVVIWIGGQRE 302
Selaginella_moellendorffii   178 GIGLPSQDRCKPESPSTFKL-LRDYIVLIASSYNLKQPA---SAGYLFWKGVYALLVGLAYCVILTGVTKLSSWFQFKVVDPGH--RNFIDVLVFE------------IEDFGTVVGNYI 279
Physcomitrella_patens        189 ELTDRLVGNCSSNELLKPNLIFGQFQTLIAHQIMMFLSSS--KTGMMFWRTALSVGLGFCYSVIVTAFTKLASFIRFRVFVA--DELSEVDILYFEAHLQALFLFVASMFSFFWLDADYE 304

                                      370       380       390       400       410       420       430
Arabidopsis_thaliana         297 KILFPNA--------------------------------------------------------------------- 303
Capsella_rubella             298 KILFPKA--------------------------------------------------------------------- 304
Camelina_sativa              298 KILFPKA--------------------------------------------------------------------- 304
Eutrema_salsugineum          293 KILFPKA--------------------------------------------------------------------- 299
Brassica_napus               288 KILFPKA--------------------------------------------------------------------- 294
Dendrobium_catenatum         132 KIIGLRQLDINSNM-------------------------------------------------------------- 145
Nicotiana_tabacum            289 RRIGLPEIAVHYSM-------------------------------------------------------------- 302
Vigna_radiata                292 KRLGLVDIFSFPKL-------------------------------------------------------------- 305
Glycine_max                  290 KRLGLMDIFRLPKS-------------------------------------------------------------- 303
Solanum_tuberosum            288 RRIGLPEIAVFYSM-------------------------------------------------------------- 301
Solanum_lycopersicum         288 RRIGLPEIAVYYSM-------------------------------------------------------------- 301
Solanum_pennellii            288 RRIGLPEIAVYYSM-------------------------------------------------------------- 301
Capsicum_annuum              284 RRIGLPEIAVYYST-------------------------------------------------------------- 297
Phaseolus_vulgaris           292 KRLGLMDIFSFPKL-------------------------------------------------------------- 305
Triticum_urartu              270 KMVGLKELVLDFKLLERFL--------------------------------------------------------- 288
Lupinus_angustifolius        289 KRLGLVDIFQFLKS-------------------------------------------------------------- 302
Medicago_truncatula          294 KRLGLADIFRFPIL-------------------------------------------------------------- 307
Hordeum_vulgare              295 KMVGLKELVLDLKLMERFL--------------------------------------------------------- 313
Oryza_sativa                 291 KMIGLKELLLDYEIMERFL--------------------------------------------------------- 309
Zea_mays                     292 KFIGLKELLSDSEVMERFF--------------------------------------------------------- 310
Sorghum_bicolor              292 KFIGLKELLSDSELMERFF--------------------------------------------------------- 310
Phalaenopsis_equestris       288 KLIGFKHLYINSDL-------------------------------------------------------------- 301
Picea_sitchensis             303 EFSRIYQLFFRGYLEGSRSEKW----WDNLEKGLKSR--------------------------------------- 335
Selaginella_moellendorffii   280 ---HLKQPVIG----------------------------------------------------------------- 287
Physcomitrella_patens        305 QHLDKVIEHFL-ISMHKLTTKAIKYCPSDLSNCLQQTRTNLAGTRNTESNRTRIIKQPLQSTILYSAPPPWHCPTA 379
```

Supplementary Figure 2. Alignment of amino acid sequences of Solyc08g075770 and similar sequences in other plants. Sequences were retrieved from BLASTP hits of Solyc08g075770 and aligned against plants from several lineages including moss, lycophyte, gymnosperm, Orchidaceae, Poaceae, Solanaceae, Brassicaceae, and Fabaceae. The intensity of blue colour indicates the degree of conservation or sequence identity: the darker the blue the more conserved the sequences. Sequences shaded with black are the predicted phosphorylation site motifs and sequences shaded with light grey are the predicted glycosylation sites.

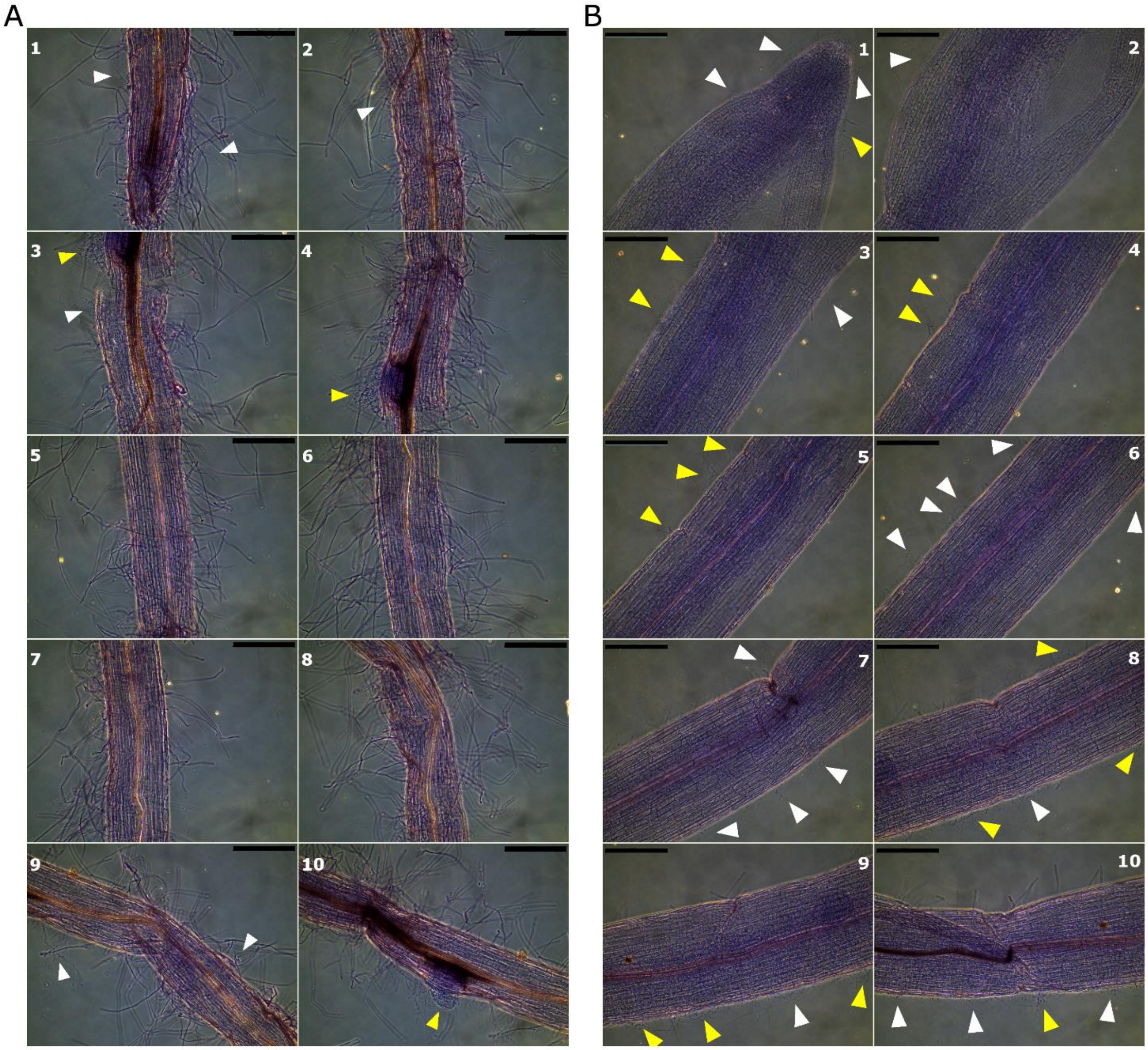

Supplementary Figure 3. Root colonisation by *Fusarium oxysporum* f. sp. *lycopersici* (*Fol*) race 3 observed by phase-contrast microscopy at 24 h after inoculation. Roots were scanned at ten overlapping zones starting from root tips and moving towards the crown. Numbers in each panel denote the sequential root zones. Light or mild colonisation is shown by white arrows whereas heavy colonisation is shown by yellow arrows. **A**, Colonisation of roots of 26AK11, a putative complemented *rmc* and **B**, root colonisation in CRISPR02, a putative knock-out 76R. Scale bars = 200 μm.

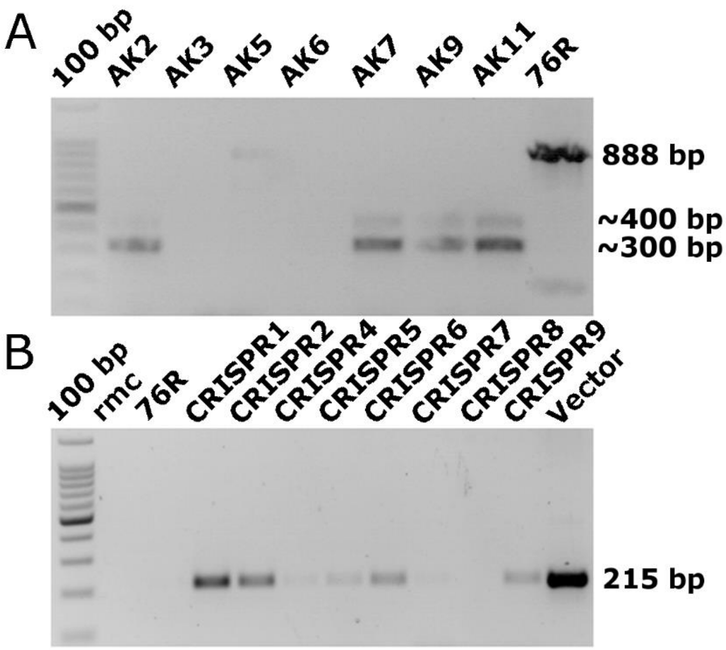

Supplementary Figure 4. Preliminary screening by PCR of putative complemented *rmc* and CRISPR-knocked-out plants. **A**, PCR amplification of *Solyc08g075770* coding sequence from several putative complemented *rmc* plantlets and 76R using primers AKCDS4-F and AKCDS4-R. These primers should amplify 174 bp fragment of *Solyc08g075770* coding sequence and 888 bp fragment of genomic DNA. The 888 bp fragment of *Solyc08g075770* was amplified from 76R, however non-specific bands were amplified form the putative complemented *rmc* which have sizes approximately 300 and 400 bp. **B**, PCR amplification of Cas9 gene in several putative knock-out mutants of 76R. 76R and *rmc* were also included. Cas9 gene was amplified using primers Cas9F1 and Cas9R1 that will amplify a 215 bp fragment. No fragments were amplified from 76R and *rmc*.

76R (forward primer)

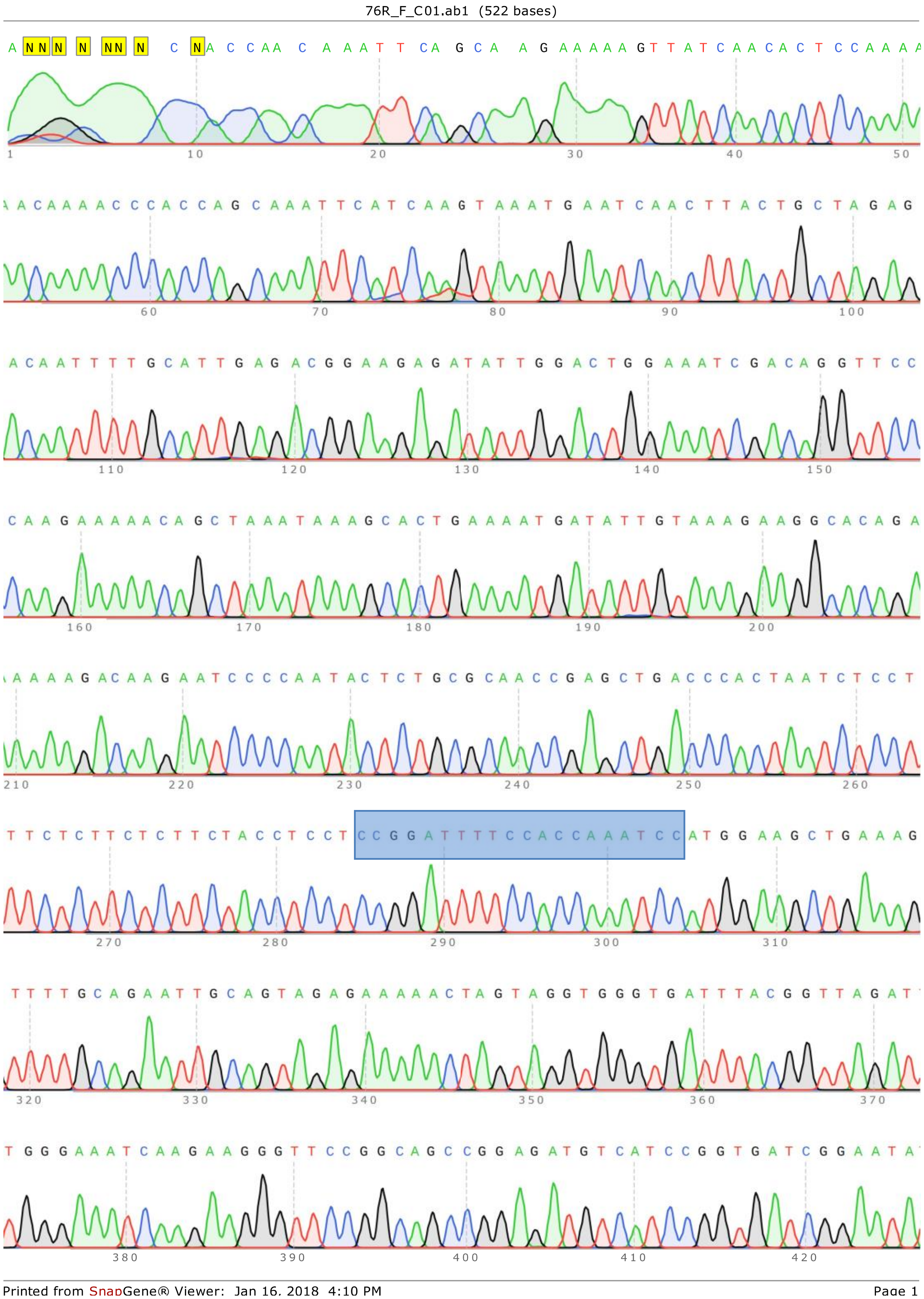

76R (reverse primer)

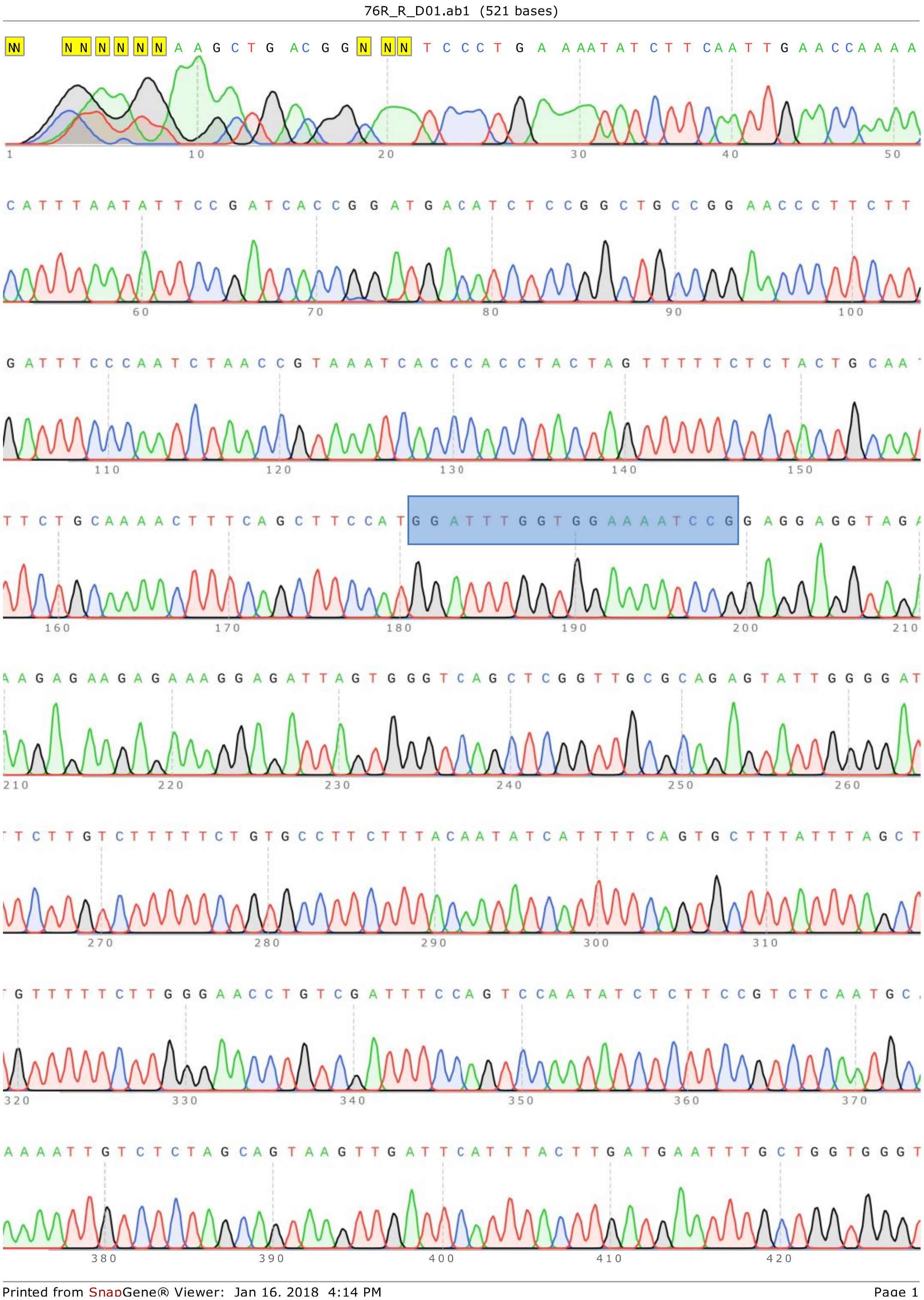

CRISPR1 (forward primer)

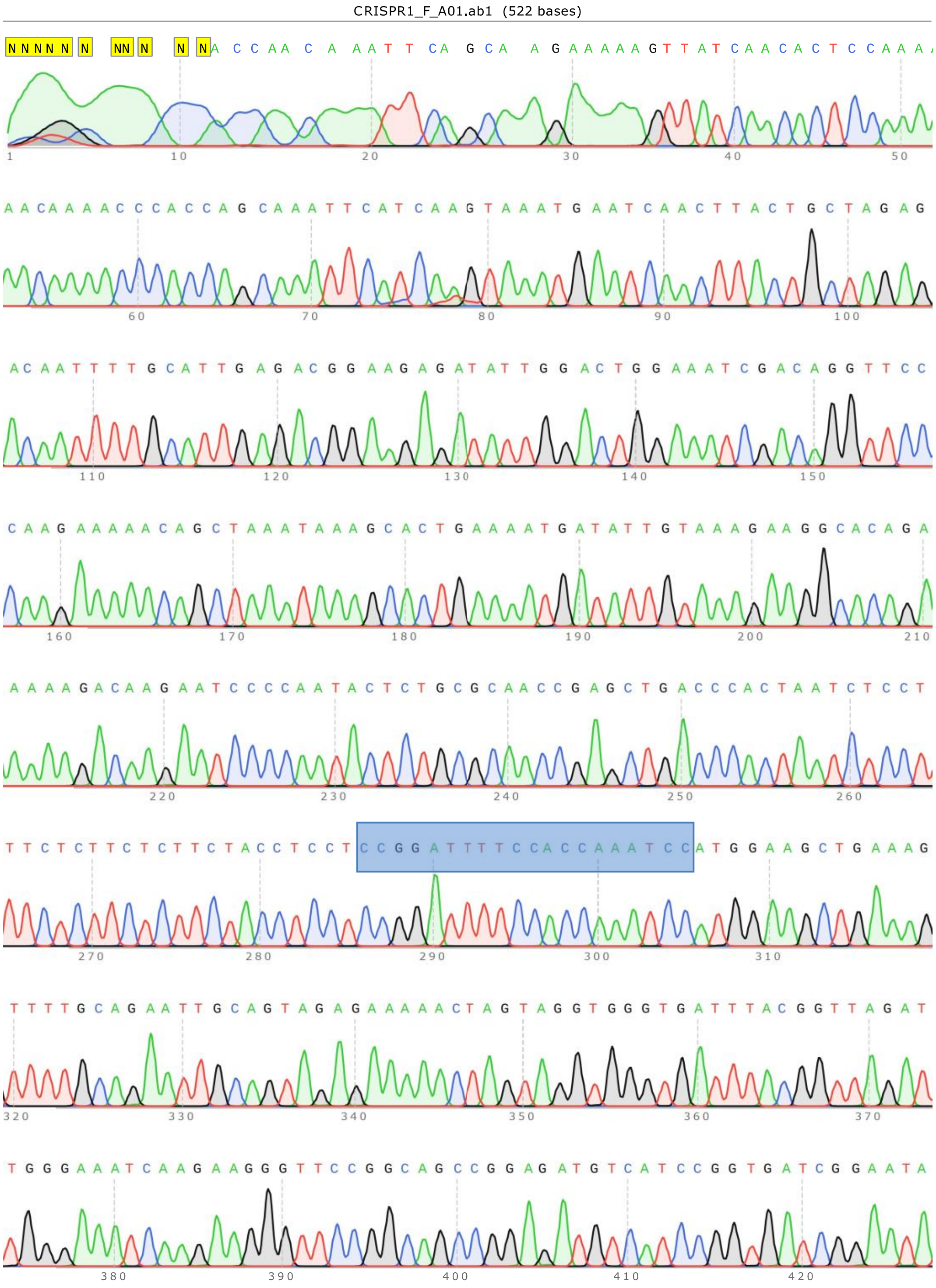

Printed from SnapGene® Viewer: Jan 16, 2018 4:18 PM Page 1

CRISPR1 (reverse primer)

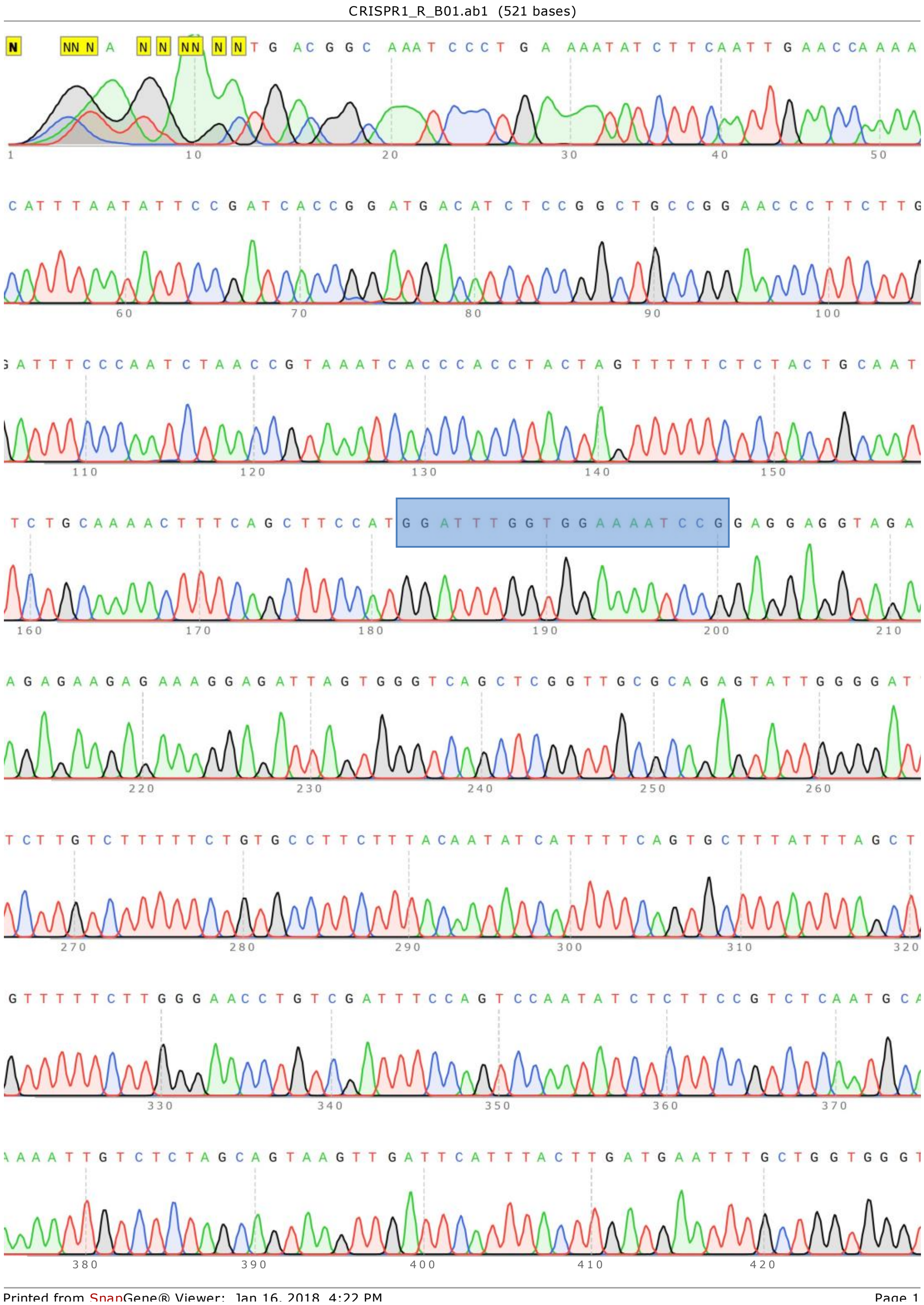

CRISPR2 (forward primer)

CRISPR2_F_A02.ab1 (521 bases)

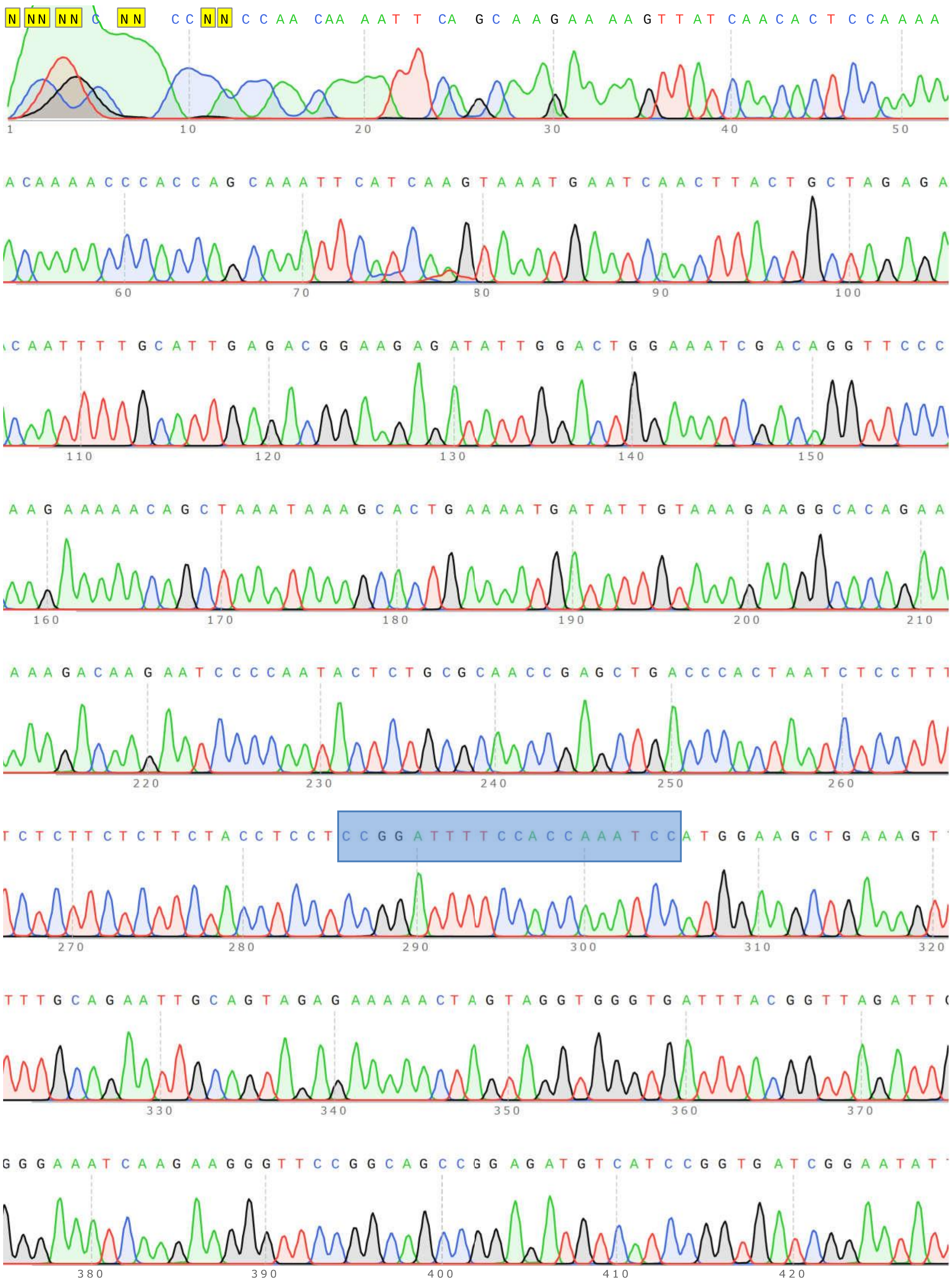

Printed from SnapGene® Viewer: Jan 19, 2018 1:48 PM

CRISPR2 (reverse primer)

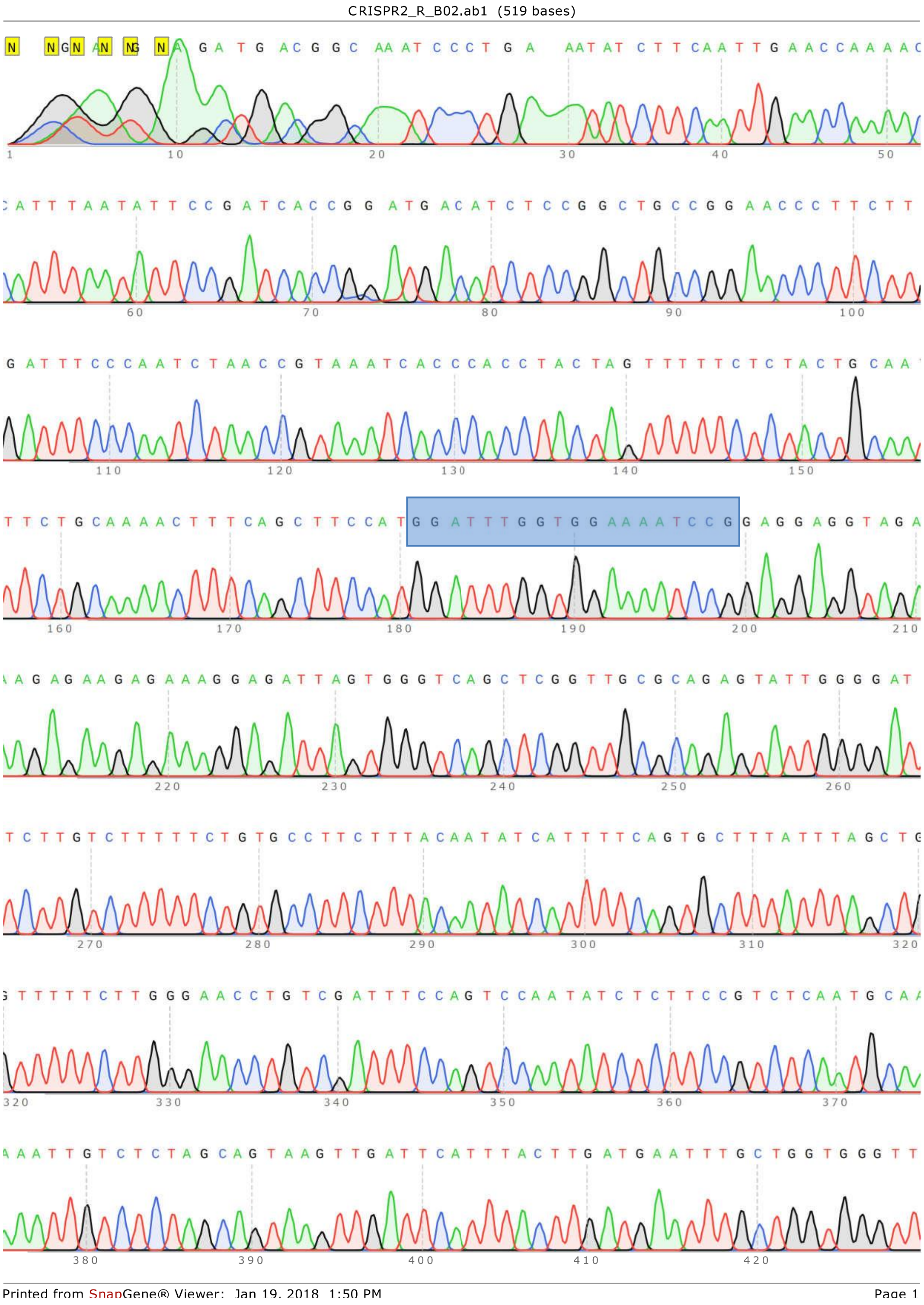

CRISPR6 (forward primer)

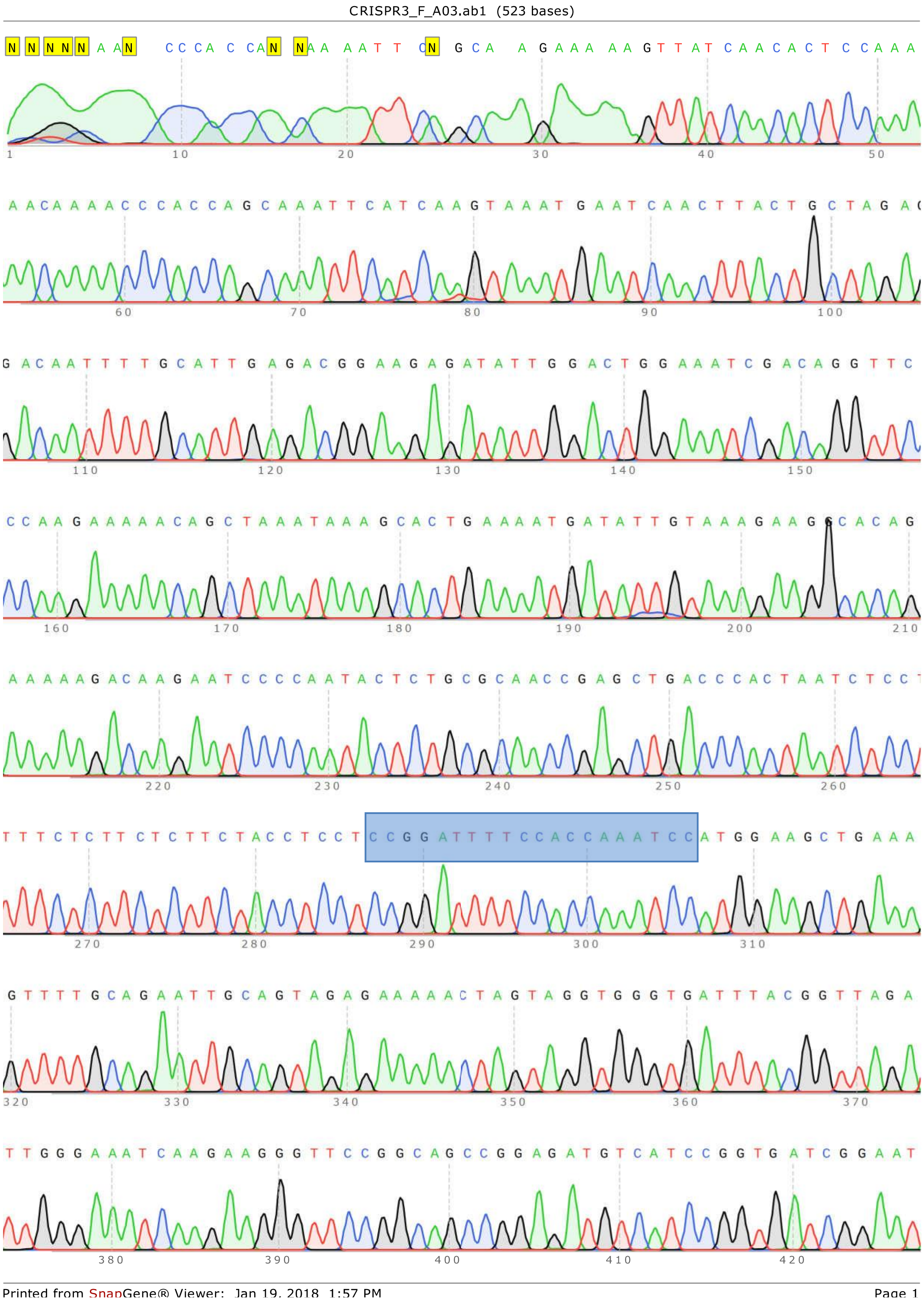

CRISPR6 (reverse primer)

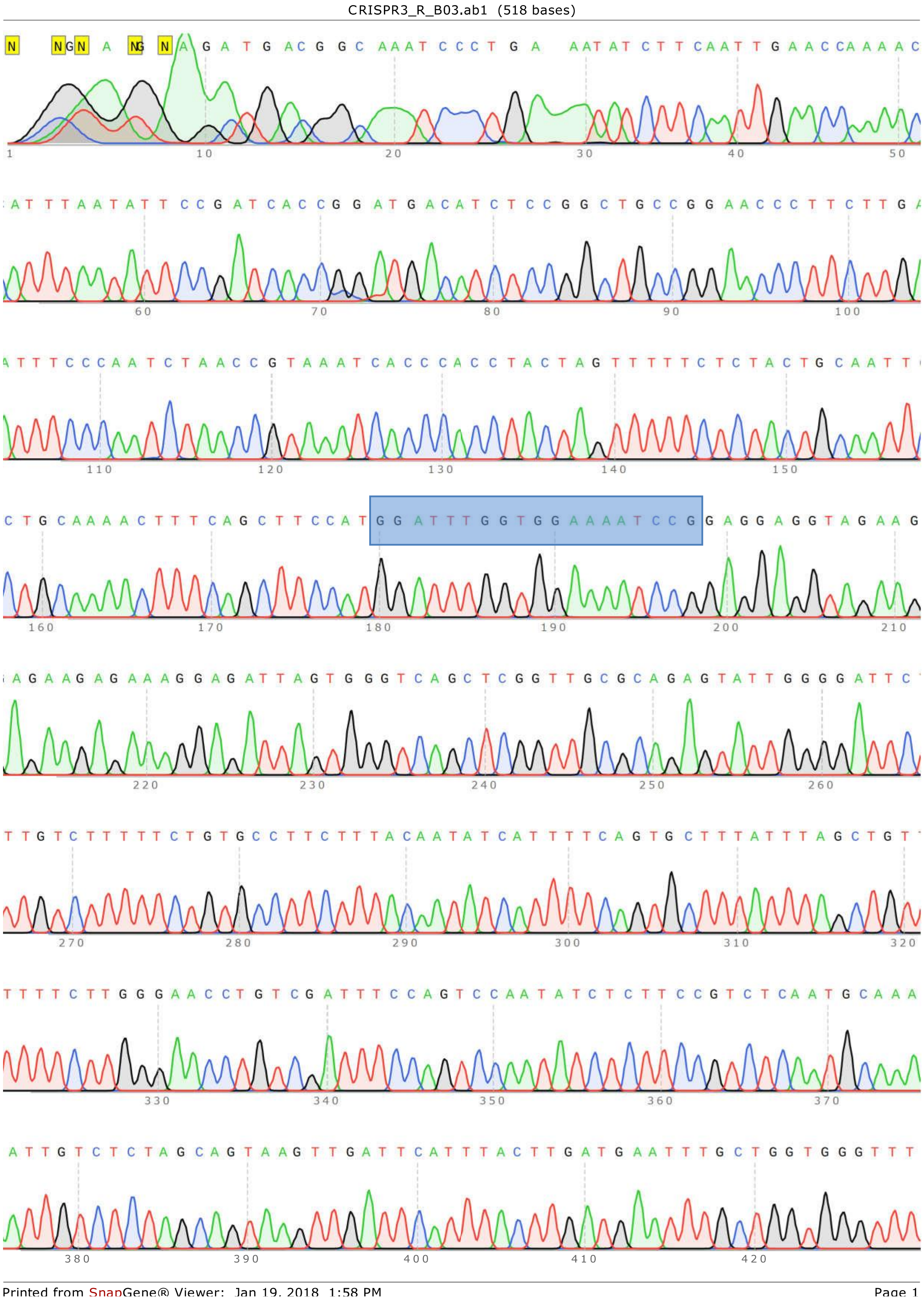

Supplementary Figure 5. Sanger sequencing of tomato 76R and plantlets of putative CRISPR-knocked-out materials. Sequence of single guide RNA (sgRNA) is shaded blue.
